# Past, Present, and Future of Viral Hepatitis C in Japan

**DOI:** 10.5005/jp-journals-10018-1166

**Published:** 2016-07-09

**Authors:** Hiroshi Yatsuhashi

**Affiliations:** Clinical Research Center, National Hospital Organization, Nagasaki Medical Center, Nagasaki, Japan

**Keywords:** Hepatitis C virus, Interferon-free therapy, Japan, Liver cancer.

## Abstract

Of all the industrialized countries of the world, Japan has the highest rate of hepatitis C. It also has 1 of the oldest and most varied histories of hepatitis C in the world among the industrialized modern nations. Hepatitis C and its complications are the leading cause of liver cancer in Japan. Japan has the highest rate of liver cancer among the industrialized countries. From 2004 to 2014, PegIFN/RBV treatment was the mainstream of hepatitis C treatment. In 2014, the first interferon-free therapy was approved in Japan. Subsequently, other interferon-free therapies have been approved. Hepatitis C virus disinfection in hepatitis C patients in Japan has become possible with a probability of 96% or more.

**How to cite this article:**

Yatsuhashi H. Past, Present, and Future of Viral Hepatitis C in Japan. Euroasian J Hepato-Gastroenterol 2016;6(1):49-51.

## HISTORY OF HCV IN JAPAN

Alan Franciscus, who is the editor in chief of the hepatitis C virus (HCV) Advocate website, introduced the history of HCV in Japan.^1^ Modern medicine and public health came early to Japan in the late 1800s. In the early 1900s, the discovery of the hypodermic needle and a drug to treat schistosomiasis would transmit hepatitis C throughout Japan. Schistosomiasis is a disease caused by a worm that lives in water snails. When people wade in water for agricultural work, the worm enters their body and lays eggs. The eggs hatch and travel to the liver. Schistosomiasis causes damage to the liver, the gastrointestinal system, kidneys, and genitals. It can, over time, cause death. In some parts of the world, it is considered as deadly as malaria. The first treatment developed to treat schistosomiasis consisted of multiple intravenous injections of antimony sodium tartrate. By the 1970s there were approximately 10 million intravenous injections given to people in Japan.^2,3^ The treatment of schistosomiasis was the beginning of the HCV epidemic in Japan. The injections were given with used or unsterile hypodermic needles.

Nagai Nagyoshi discovered methamphetamine in 1893. Dr. Ogata Akira was able to synthesize it into crystalline meth in 1919. Widespread use of methamphetamine use did not begin until World War l, when it was used as an injectable treatment for asthma. The large-scale use came later during World War II, when it was prescribed as an oral and injectable stimulant for tired soldiers, pilots, and ammunition workers during the war. After the war methamphetamine was prescribed for general postwar trauma. In 1949, Japan banned the manufacture of methamphetamine, but illegal methamphetamine use continued as did the hepatitis C epidemic.^4^

## CURRENT STATUS OF HCV INFECTION IN JAPAN

Of all the industrialized countries of the world, Japan has the highest rate of hepatitis C (caused by HCV). It also has 1 of the oldest and most varied histories of hepatitis C in the world among the industrialized modern nations. Hepatitis C and its complications are the leading causes of liver cancer in Japan. Japan has the highest rate of liver cancer among the industrialized countries. Hepatitis C virus is the 4th leading cause of death among Japanese men and the 5th leading cause of mortality among Japanese women. Of the 1.2 million people living with HCV infection in Japan, approximately 70% have HCV genotype 1b and 30% have genotype 2a/b. Further, a significant number of patients with HCV infection in Japan are over the age of 65, leading to more disease-related complications and a decreased likelihood of tolerating interferon-based therapies, the historical standard of care for treating HCV. Japan has a multilayered health care system. Many people can get health care insurance through their employer or the national health care system. The government system covers about 70% and the patient covers the remaining 30%.

## PROGRESS AND FUTURE OF HEPATITIS C THERAPY IN JAPAN

From 2004 to 2014, PegIFN/RBV treatment was the mainstream of hepatitis C treatment. In 2014, the first interferon-free therapy was approved in Japan. Subsequently, other interferon-free therapies have been approved, which are listed below:

- September 2014 – Asunaprevir and Daclatasvir – HCV1
- May 2015 – SOVALDI® (sofosbuvir)/RBV – HCV2
- September 2015 – HARVONI – HCV1
- December 2015 – VIEKIRA PAK – HCV1

The HCV disinfection in hepatitis C patients in Japan has become possible with a probability of 96% or more.

## DACLATASVIR AND ASUNAPREVIR COMBINATION THERAPY FOR HCV1 IN JAPAN

All oral combinations of direct-acting antivirals may improve the efficacy and safety outcomes for patients with HCV infection, particularly those who are poor candidates for interferon/ribavirin-based regimens.

In July 2014, the Japanese Ministry of Health, Labor and Welfare (MHLW) approved Daklinza® (daclatasvir), a potent, pan-genotypic NS5A replication complex inhibitor (*in vitro*), and Sunvepra® (asunaprevir), an NS3/4A protease inhibitor. The Daklinza + Sunvepra dual regimen is Japan’s first all-oral, interferon- and ribavirin-free treatment regimen for patients with genotype 1 chronic HCV infection, including those with compensated cirrhosis.

In 2014, Kumada et al^5^ reported the results of a phase 3, open-label study to assess the efficacy and safety of daclatasvir and asunaprevir combination therapy. In this clinical trail, 135 interferon-ineligible/intolerant and 87 nonresponder patients with chronic HCV genotype 1b infection were enrolled at 24 centers in Japan. Patients received daclatasvir 60 mg once daily plus asunaprevir 100 mg twice daily for 24 weeks. The primary endpoint was sustained virologic response 24 (SVR_24_) weeks after treatment. Sustained virologic response_24_ was achieved by 87.4% of interferon-ineligible/intolerant patients and 80.5% of nonresponder (null and partial) patients; rates were similar in cirrhosis (90.9%) and non-cirrhosis (84.0%) patients, and in patients with *IL28B* CC (84.5%) or non-CC (84.8%) genotypes. Fourteen patients in each group (12.6%) discontinued dual therapy, mainly due to adverse events or lack of efficacy. Nine nonresponder patients received additional treatment with pegIFN/RBV per protocol-defined criteria. The rate of serious adverse events was low (5.9%) and varied among patients. The most common adverse events were nasopharyngitis, increased alanine aminotransferase (ALT) and aspartate aminotransferase (AST), headache, diarrhea, and pyrexia. They concluded that interferon-free, ribavirin-free, all-oral therapy with daclatasvir and asunaprevir for 24 weeks is well tolerated and can achieve a high rate of SVR in patients with HCV genotype 1b who were ineligible and intolerant or had not responded to prior interferon-based therapy.

## SOFOSBUVIR TREATMENT FOR HCV2 AND HCV1 IN JAPAN

In May 2015, the Japanese MHLW approved Sovaldi® (sofosbuvir), a once-daily nucleotide analog polymerase inhibitor, for the suppression of viremia in patients with genotype 2 chronic HCV infection with or without compensated cirrhosis. Sovaldi is indicated for use in combination with ribavirin (RBV) for 12 weeks. Sovaldi (in combination with RBV) is the first all-oral, interferon-free treatment regimen for genotype 2 HCV infection.

In 2014, M. Omata et al^6^ reported the results of a phase 3, open-label study to assess the efficacy and safety of an all-oral combination of the NS5B polymerase inhibitor sofosbuvir and RBV in patients with chronic genotype 2 HCV infection. They enrolled 90 treatment-naive and 63 previously treated patients at 20 sites in Japan. All patients received sofosbuvir 400 mg plus RBV (weight-based dosing) for 12 weeks. The primary endpoint was sustained virologic response at 12 SVR_12_ weeks after therapy. Of the 153 patients enrolled and treated, 60% had HCV genotype 2a, 11% had cirrhosis, and 22% were 65 years or older. Overall, 148 patients (97%) achieved SVR_12_. Of the 90 treatment-naıve patients, 88 (98%) achieved SVR_12_, and of the 63 previously treated patients, 60 (95%) achieved SVR_12_. The rate of SVR_12_ was 94% in patients with cirrhosis and in those aged 65 and older. No patients discontinued study treatment due to adverse events. The most common adverse events were nasopharyngitis, anemia, and headache. They concluded that 12 weeks of sofosbuvir and RBV resulted in high rates of SVR_12_ in treatment-naive and previously treated patients with chronic genotype 2 HCV infection. The treatment was safe and well tolerated by patients, including the elderly and those with cirrhosis.

In July 2015, the Japanese MHLW approved Harvoni® (ledipasvir 90 mg/sofosbuvir 400 mg), the first once-daily single-tablet regimen for the treatment of chronic hepatitis C genotype 1 infection in adults. Harvoni combines the NS5A inhibitor ledipasvir with the nucleotide analog polymerase inhibitor sofosbuvir, approved by the MHLW under the trade name Sovaldi. Harvoni is indicated for the suppression of viremia in patients with genotype 1 chronic hepatitis C virus (HCV) infection with or without compensated cirrhosis, with the treatment duration of 12 weeks.

Mizokami et al^7^ reported the results of an open-label study to assess the efficacy and safety of Harvoni in patients with chronic genotype 1 HCV infection in Japan. In this randomized, open-label study, we enrolled patients from 19 clinical Japanese centers. Patients were randomly assigned (1:1) to receive either ledipasvir (90 mg) and sofosbuvir (400 mg) or ledipasvir, sofosbuvir, and RBV orally once daily for 12 weeks. A total of 341 patients were randomly assigned to treatment groups and received at least 1 dose of study treatment. Sustained virologic response_12_ was achieved in all 171 (100%) patients (83 of 83 treatment naive and 88 of 88 treatment experienced) receiving ledipasvir–sofosbuvir and 167 (98%) of 170 patients (80 of 83 treatment naive and 87 of 87 treatment experienced) receiving ledipasvir–sofosbuvir plus RBV. Of the 76 patients with baseline NS5A-resistant variants, 75 (99%) achieved SVR_12_. 2 (1.2%) of 170 patients in the ledipasvir–sofosbuvir plus RBV group discontinued treatment because of adverse events. The most common adverse events were nasopharyngitis (29.2%), headache (7.0%), and malaise (5.3%) in patients receiving ledipasvir–sofosbuvir, and nasopharyngitis (23.5%), anemia (13.5%), and headache in those receiving ledipasvir–sofosbuvir plus RBV (8.8%). They concluded that the efficacy, tolerability, and absence of drug–drug interactions of ledipasvir–sofosbuvir suggest that it could be an important option for the treatment of genotype 1 HCV in Japanese patients.
